# Management of Graves Disease With Co-Existing Duffy-Null Phenotype

**DOI:** 10.1210/jcemcr/luaf157

**Published:** 2025-07-28

**Authors:** Grace M Ferri, Cassandra Chua, Alexa R Trovato, Alejandro Campos, Mark Sloan, Shirin Haddady

**Affiliations:** Section of General Internal Medicine, Department of Medicine, Boston Medical Center, Boston, MA 02118, USA; Section of General Internal Medicine, Department of Medicine, Boston Medical Center, Boston, MA 02118, USA; Section of General Internal Medicine, Department of Medicine, Boston Medical Center, Boston, MA 02118, USA; Section of General Internal Medicine, Department of Medicine, Boston Medical Center, Boston, MA 02118, USA; Section of Hematology/Oncology, Boston Medical Center, Boston, MA 02118, USA; Section of Endocrinology, Boston Medical Center, Boston, MA 02118, USA

**Keywords:** antithyroid drugs, neutropenia, Graves disease, hyperthyroidism, agranulocytosis

## Abstract

Clinicians should obtain baseline white blood cell differential among those in whom antithyroid drugs (ATD) will be initiated, as patients may have neutropenia prior to initiation of ATD. We report the case of a 37-year-old male individual who had emigrated from Saudi Arabia and was found to have Graves disease. At diagnosis, the patient was leukopenic and neutropenic. Prior neutrophil counts had ranged from moderate neutropenia to normal. The patient was found to have the Duffy-null phenotype on red blood cell antigen typing, which is characterized by a clinically insignificant lower absolute neutrophil count (ANC) relative to commonly used reference ranges and no known increased risk of infection. Despite his baseline neutropenia, the patient received a standard weight-based dose of methimazole. ATD should not be withheld from patients with the Duffy-null phenotype. These patients have fewer circulating neutrophils without an increased risk of infection.

## Introduction

Red blood cell antigen typing facilitates the diagnosis or exclusion of Duffy-null associated neutrophil count (DANC) [1, 2]. The Duffy-null phenotype is present in 80% to 100% of people from Sub-Saharan Africa relative to <1% of people of Asian or Caucasian descent [1]. In the United States, nearly 67% of individuals of African or Middle Eastern ancestry have the Duffy-null phenotype [2] Persons with DANC have a clinically insignificant lower absolute neutrophil count (ANC) relative to commonly used reference ranges and harbor no increased risk of infection [3]. Although total body neutrophils are adequate in number, the number of neutrophils marginating to the periphery in these individuals is lower. As a result, those with DANC are often subjected to unnecessary or invasive testing, including bone marrow biopsies; exclusion from clinical trials; or premature discontinuation of drugs including chemotherapies and immunomodulatory agents [4]. Phenotyping of the Duffy antigen receptor for chemokines (DARC) expressed on red blood cells is readily available in most blood banks [5].

## Case Presentation

A 37-year-old male individual with a history of intravenous drug use presented to the emergency department with chest pain. Approximately 10 years prior to presentation, the patient had emigrated from Saudi Arabia to the United States. The patient denied any personal or family history of autoimmune or skin conditions.

## Diagnostic Assessment

On examination, the patient was found to be febrile to 101° F, tachycardic to 120 beats/minute, and tachypneic to 22 breaths/minute with blood pressure 127/70 millimeters of mercury and oxygen saturation normal on room air. Examination revealed diffuse enlargement of thyroid gland. Laboratory studies were notable for white blood cell (WBC) count 2.0 K/μL (International System of Units [SI]: 2.0 × 10^9^/L) (reference range, 4.0-11.0 K/μL [SI: 4.0 - 11.0 × 10^9^/L]) with an ANC 0.4 K/μL (SI: 0.4 × 10^9^/L) (reference range, 1.8-7.0 K/μL [SI: 1.8-7.0 × 10^9^/L]); hemoglobin 11.3 g/dL (SI: 113 g/L); normal platelet count; normal liver function tests; normal serial troponin I level; thyroid stimulating hormone <0.01 uIU/mL (SI: < 0.01 IU/L) (reference range, 0.35-4.9 μIU/mL [SI: 0.4-4.5 IU/L]) with free thyroxine 3.84 ng/dL (SI: 49.4 pmol/L) (reference range, 0.8-1.8 ng/dL [SI: 10.3-23.2 pmol/L]); and thyroid stimulating immunoglobulins 364% (reference <140%). Peripheral blood smear with normocytic, normochromic red blood cells with occasional acanthocytes and target cells. During the year prior to admission, ANC range for the patient had been 0.6 to 6.0 K/μL. Antibodies for human immunodeficiency virus and hepatitis B and C were negative.

## Treatment

Despite his moderate-severe neutropenia, the patient received methimazole at approximately 0.25 mg/kg/day (20 mg oral daily).

## Outcome and Follow-Up

The patient received red blood cell antigen typing revealing the Duffy-null phenotype, which predisposes him to a lower baseline ANC. Identifying the Duffy-null phenotype in this patient established a baseline neutrophil count, prevented unnecessary antithyroid drug (ATD) dose modification, and lowered the index of suspicion for future episodes of neutropenia due to drug-induced agranulocytosis. Within 90 days after starting methimazole, his ANC range was 1.2 to 2.0 K/μL (SI: 1.2-2.0 × 10^9^/L) and free thyroxine 1.04 ng/dL (SI: 49.4 pmol/L).

## Discussion

To our knowledge, this is the first reported case of Graves disease in a patient found to have the Duffy-null phenotype. Per the American Thyroid Association recommendations for diagnosing and managing hyperthyroidism, clinicians should obtain baseline blood testing among those in whom ATD will be initiated; these initial laboratory values should assist in the interpretation of future studies while on therapy [6]. The guidelines note that those of African American descent are frequently found to have low WBC counts, herein alluding to the Duffy-null phenotype, and that ANC values between 1 and 2 K/μL should not preclude treatment initiation [6]. While no discrete evidence exists to correlate preexisting neutropenia to ATD-related complications, the American Thyroid Association does recommend reconsideration of ATD use among those with moderate-severe neutropenia (ANC < 1 K/μL) out of caution for drug-induced agranulocytosis [6, 7].

Agranulocytosis is diagnosed when the absolute number of granulocytes (including neutrophils, eosinophils, and basophils) falls under 500/mm^3^ [8]. The phenomenon of agranulocytosis secondary to ATD has been reported in 0.37% of patients receiving propylthiouracil and 0.35% of patients receiving methimazole [9]. Per a population-based case-control study, patients exposed to ATD were found to have greater than 50 times the risk of agranulocytosis relative to those unexposed [7]. The risk of methimazole- and propylthiouracil-induced agranulocytosis is thought to be dose-dependent, where patients on higher doses of ATD are more likely to develop agranulocytosis [10, 11]. Moreover, patients aged 65 and older have been found to have higher rates of drug-induced agranulocytosis and associated fatalities [7, 12].

In terms of monitoring, drug-induced agranulocytosis usually occurs within 90 days but can arise more than 1 year after treatment has begun [8]. Patients should be instructed to hold ATD if any signs or symptoms of agranulocytosis (including fever, sore throat, or oral ulcers) develop [8]. Clinicians should immediately evaluate the patient, obtain a WBC count with differential, and formally discontinue the ATD if granulocyte count is <1000/mm^3^ [8].

When examining bone marrow biopsies, patients exposed to ATD have been found to have (a) myeloid hypoplasia resulting in reduction of granulocyte precursors or (b) increased production of immature neutrophils (including myelocytes and metamyelocytes) at the expense of mature granulocytes [13-15]. When hypocellularity occurs, manifestations are not limited to agranulocytosis but can include thrombocytopenia or aplastic anemia [14, 16]. Those with ATD-induced agranulocytosis are susceptible to infections with *Pseudomonas aeruginosa* and thus are candidates to receive granulocyte-colony stimulating factor if deemed high-risk [17, 18].

Studies suggest that approximately 10% of patients with Graves disease initially present with neutropenia, usually to a mild or moderate degree [19]. When patients with Graves disease and associated mild-moderate neutropenia receive ATD, their neutropenia generally resolves without subsequent decreases in ANC or development of agranulocytosis [19]. Mild neutropenia is thus not considered a contraindication to the initiation of thionamides in any patients.

Patients like ours with both DANC and Graves disease have not been studied. However, complete recovery of neutrophil count would not similarly be expected to occur after ATD in these patients, as the underlying mechanism of DANC involves reduced neutrophil margination to the periphery. Thus, pretreatment testing for alternate causes of baseline neutropenia remains prudent among patients with newly diagnosed Graves disease.

The differential diagnosis for agranulocytosis, leukopenia, or neutropenia among patients diagnosed with Graves disease hinges on chronicity. For patients with neutropenia that predates endocrinologic symptoms, congenital, autoimmune, or dietary causes warrant consideration [20]. If the Duffy-null genotype is not identified, patients may benefit from anti-neutrophil antibody testing, as systemic autoimmune diseases can often co-occur with hyperthyroidism [21]. For patients with acute neutropenia or neutropenia of unknown chronicity, a hematopathologist should evaluate the peripheral blood smear for WBC abnormalities or blasts suggesting malignancy.

Although the American Thyroid Association does recommend reconsideration of ATD use among those with moderate-severe neutropenia out of caution for drug-induced agranulocytosis, we advocate for a trial of ATD among patients known to have the Duffy-null phenotype, as illustrated in our patient case. Given the pathophysiology of reduced neutrophil margination to the periphery in the Duffy-null phenotype, a preexisting mild, moderate, or severe neutropenia should not affect outcomes in this population. Moreover, interval fluctuations in ANC should not prompt ATD dose modification in the absence of symptoms. Future studies including patients with DANC receiving ATD would further assist in clinical decision-making and pharmacologic recommendations.

## Learning Points

- The differential diagnosis for neutropenia is broad but includes both Graves disease and DANC.
- Mild neutropenia is not a contraindication to antithyroid drugs.
- No evidence exists to suggest that baseline neutropenia increases the risk of complications from ATD.

## Data Availability

Original data generated and analyzed during this study are included in this published article.
